# *Desulfovibrio* bacteria enhance alpha-synuclein aggregation in a *Caenorhabditis elegans* model of Parkinson’s disease

**DOI:** 10.3389/fcimb.2023.1181315

**Published:** 2023-05-01

**Authors:** Vy A. Huynh, Timo M. Takala, Kari E. Murros, Bidhi Diwedi, Per E. J. Saris

**Affiliations:** ^1^ Department of Microbiology, Faculty of Agriculture and Forestry, University of Helsinki, Helsinki, Finland; ^2^ Adjunct Professor of Neurology, University of Eastern Finland, Kuopio, Finland; ^3^ University of Padova, Padova, Italy

**Keywords:** Parkinson’s disease, gut *Desulfovibrio* bacteria, alpha-synuclein (alpha-syn), *C. elegans*, hydrogen sulfide, lipopolysaccharides, curli-producing *E. coli*, magnetite

## Abstract

**Introduction:**

The aggregation of the neuronal protein alpha-synuclein (alpha-syn) is a key feature in the pathology of Parkinson’s disease (PD). Alpha-syn aggregation has been suggested to be induced in the gut cells by pathogenic gut microbes such as *Desulfovibrio* bacteria, which has been shown to be associated with PD. This study aimed to investigate whether *Desulfovibrio* bacteria induce alpha-syn aggregation.

**Methods:**

Fecal samples of ten PD patients and their healthy spouses were collected for molecular detection of *Desulfovibrio* species, followed by bacterial isolation. Isolated *Desulfovibrio* strains were used as diets to feed *Caenorhabditis elegans* nematodes which overexpress human alpha-syn fused with yellow fluorescence protein. Curli-producing *Escherichia coli* MC4100, which has been shown to facilitate alpha-syn aggregation in animal models, was used as a control bacterial strain, and *E. coli* LSR11, incapable of producing curli, was used as another control strain. The head sections of the worms were imaged using confocal microscopy. We also performed survival assay to determine the effect of *Desulfovibrio* bacteria on the survival of the nematodes.

**Results and Discussion:**

Statistical analysis revealed that worms fed *Desulfovibrio* bacteria from PD patients harbored significantly more (*P*<0.001, Kruskal-Wallis and Mann-Whitney U test) and larger alpha-syn aggregates (*P*<0.001) than worms fed *Desulfovibrio* bacteria from healthy individuals or worms fed *E. coli* strains. In addition, during similar follow-up time, worms fed *Desulfovibrio* strains from PD patients died in significantly higher quantities than worms fed *E. coli* LSR11 bacteria (*P*<0.01). These results suggest that *Desulfovibrio* bacteria contribute to PD development by inducing alpha-syn aggregation.

## 1 Introduction

Parkinson’s disease (PD) is a common age-related neurodegenerative disorder that primarily hinders movement. Despite more than 200 years of research, the essential etiopathogenetic mechanisms of Parkinson’s disease have remained enigmatic. Genetic, environmental, and lifestyle factors evidently play some role in the disease pathogenesis (Jankovic and Tan, 2020), and it is likely that bacteria and viruses take part in the pathogenesis of PD (Murros et al., 2021; Smeyne et al., 2021; Murros, 2022). Accumulation of alpha-synuclein (alpha-syn) protein in the form of Lewy bodies and Lewy neurites is the neuropathological hallmark of Parkinson’s disease. Post-mortem analyses on PD neuropathology have revealed alpha-syn depositions not only in the brain, but also in the spinal cord, in the autonomous nerves, in the peripheral plexuses of the enteric nervous system and in the nerves of the skin, submandibular gland, and myocardial tissue (Wakabayashi et al., 1988; Braak et al., 1999; Braak et al., 2007; Beach et al., 2010; Gelpi et al., 2014; Borghammer et al., 2021; Isonaka et al., 2022). Importantly, aggregation of alpha-syn has also been found in human gastrointestinal tract such as colon (Visanji et al., 2015; Chung et al., 2016), and gastric mucosa (Sánchez-Ferro et al., 2015). A recent, eventually the first *in vivo* study on duodenal biopsy specimens looking for alpha-syn pathology in PD patients and healthy individuals showed marked immunoreactivity for aggregated alpha-syn in every studied specimen of PD patient whereas the corresponding immunoreactivity was either absent or only barely detectable in controls (Emmi et al., 2023). Neuropathological observations and analyses have justified a hypothesis that a pathogen in the gut may induce alphasyn aggregation, which then spreads *via* the vagal nerve to the central nervous system in the brain (Braak et al., 2006). Alpha-syn is expressed in enteroendocrine cells, which are neuron-like cells residing with the greatest frequency in the small intestine (Sjölund et al., 1983) and connected to the enteric alpha-syn containing nerves (Chandra et al., 2017). Based on this finding, Chandra et al. have proposed that alpha-syn aggregation is likely initiated in the enteroendocrine cells by the action of an intestinal pathogen; alpha-syn aggregates then spread in a prion-like manner from the gut to the brain *via* the neural circuit (Chandra et al., 2017).

Accumulated studies in different animal models have provided further insight into the potential role of gut bacteria in PD pathology. In human alpha-syn overexpressing rats and mice, introduction of *Escherichia coli* that produces curli (an extracellular amyloid fiber) *via* the oral route led to accelerated alpha-syn aggregation and intestinal and motor deficits (Chen et al., 2016; Sampson et al., 2020). Increased alpha-syn aggregation was also observed in a *Caenorhabditis elegans* model that expresses human alpha-syn after exposure to curli-producing *E. coli* (Chen et al., 2016). Intranasal exposure to lipopolysaccharide, an endotoxin produced by Gram-negative bacteria, promoted alpha-syn aggregation in the olfactory bulb and substantia nigra of mice and triggered inflammatory responses and PD-like behavioral issues (Niu et al., 2020). Although an association of curli-producing *E. coli* with PD has remained speculative, these observations suggest that gut microbial metabolites, components, or products may indeed have a role in PD by promoting alpha-syn pathology. Anyhow, the identification of the etiologic agent initiating PD development is still incomplete.

We recently studied the association between the sulfate reducing *Desulfovibrio* bacteria (*DSV*) and PD (Murros et al., 2021). The bacteria were more prevalent and more abundant in quantity in PD patients, especially in patients with more severe disease, than in healthy individuals (Murros et al., 2021). However, it has not been investigated how these Gram-negative bacteria contribute to the development of PD, especially regarding alpha-syn pathology. Such studies are necessary, as *DSV* bacteria possess special characteristics. Specifically, they can produce hydrogen sulfide (H_2_S) (Kushkevych et al., 2019) and at least some strains can synthesize magnetite (Fe_3_O_4_) (Lovley et al., 1993). H_2_S produced by gut bacteria can have harmful effects on human cells by facilitating mitochondrial cytochrome c release, enhancing iron levels, and promoting reactive oxygen species formation, which eventually leads to alpha-syn aggregation (Murros, 2022). *DSV*-produced Fe_3_O_4_ can also play a role in aggregation as Fe_3_O_4_ nanoparticles can promote reactive oxygen species formation (Könczöl et al., 2011) and accelerate alpha-syn aggregation (Joshi et al., 2015).

In this study, we used a *C. elegans* model expressing human alpha-syn fused with yellow fluorescent protein (van Ham et al., 2008) to investigate whether *DSV* strains isolated from PD patients and healthy individuals can contribute to alpha-syn aggregation. Our results showed that *DSV* bacteria, especially patient strains, enhanced alpha-syn aggregation in the worms.

## 2 Materials and methods

### 2.1 Research subjects, sample collection, and ethical permission

The present study was a sub-study of an on-going study focusing on magnetic properties of fecal samples in Parkinson’s disease (PD). The study included 20 participants (ten PD patients and ten healthy individuals). The patients were recruited from the Neurology Outpatient Clinic of Terveystalo Healthcare, Helsinki, Finland. Healthy individuals were spouses of patients. The criteria for participant recruitment and handling of fecal samples were similar to those previously described (Murros et al., 2021). Research approval was granted by the Ethics Committee of Helsinki and the Uusimaa Health District (no. 2510/2020). Relevant regulations were followed in all procedures. Written informed consent was provided by all participants.

### 2.2 Isolation of *Desulfovibrio* bacteria

The following procedures and bacterial incubation were performed in an anaerobic workstation (Don Whitley Scientific, West Yorkshire, UK). One gram of feces was homogenized in 5 ml of Postgate broth (DSMZ medium 63). The suspension was incubated anaerobically at 37°C for 3-4 days until signs of bacterial growth appeared, which was indicated by blackening of the suspension. Bacterial growth was enriched by an additional subculture in Postgate broth. The bacterial suspension was then streaked on Postgate agar plates, followed by incubation at 37°C for 3-4 days. Single black colonies were repeatedly picked and streaked until pure single colonies were obtained. Pure cultures were analyzed with phase-contrast microscopy. Isolates with *Desulfovibrio* (*DSV*)-like cell morphology were identified by molecular techniques described below. The *DSV* isolates were cryopreserved at -75°C in Postgate broth containing 17% glycerol (VWR Chemicals, Leuven, Belgium).

### 2.3 Molecular techniques

#### 2.3.1 DNA extraction

For the detection of *Desulfovibrio* bacteria, DNA was extracted from the fecal samples using a Stool DNA Isolation Kit (Norgen Biotek Corp., Thorold, ON, Canada). For the identification of the *DSV* isolates, bacterial DNA was extracted using a MagAttract HMW DNA Kit (Qiagen GmbH, Hilden, Germany). Extracted DNA was stored at -20°C.

#### 2.3.2 Primers and PCR conditions

Bacterial 16S rRNA region was amplified using the universal primers pA and pE’, either for identification of *DSV* isolates or for use as an indicator of successful DNA extraction from feces. Specific detection of *DSV* bacteria was done using species-specific primers targeting 16S rRNA region of *D. desulfuricans*, *D. piger*, *D. fairfieldensis* and *D. vulgaris*. A pair of primers targeting [FeFe]-hydrogenase gene (*hydA*), which is present in nearly all *DSV* bacteria, was used to detect the presence of other *DSV* species. All the used primers are listed in **Table 1**.

**Table 1:** Primers used in the study.

Primers	Sequence 5’ → 3’	Target	Sources
**pA**	AGAGTTTGATCCTGGCTCAG	Bacterial 16S rRNA	Edwards et al., 1989
**pE’**	CCGTCAATTCCTTTGAGTTT		
**27K-F**	CTGCCTTTGATACTGCTTAG	16S rRNA of *D. desulfuricans*	Loubinoux et al., 2002
**27K-R**	GGGCACCCTCTCGTTTCGGAGA		
**Fair-F**	TGAATGAACTTTTAGGGGAAAGAC	16S rRNA of *D. fairfieldensis*	Loubinoux et al., 2002
**P687-R**	GATATCTACGGATTTCACTCCTACACC		
**Pig-F**	CTAGGGTGTTCTAATCATCATCCTAC	16S rRNA of *D. piger*	Loubinoux et al., 2002
**P687-R**	GATATCTACGGATTTCACTCCTACACC		
**Dv1F**	AAGACCTTCCCGAAAAGGAA	16S rRNA of *D. vulgaris*	Chakraborty et al., 2011
**Dv1R**	ACCAGAGTGCCCAGCATTAC		
***hydA*-F**	GACGTGACCATCTGGGAAGA	Periplasmic [FeFe]-hydrogenase gene of *DSV* bacteria	Murros et al., 2021
***hydA*-R**	CAGGCCATGAATTCGATGAA		

One 50 µl PCR reaction consisted of 1× Phusion Green HF buffer (Thermo Fisher Scientific, Waltham, MA, USA), 0.2 mM dNTP mix (Thermo Fisher Scientific), 0.5 µM of each primer, 1 U of Phusion High-Fidelity DNA polymerase (Thermo Fisher Scientific), and approximately 15-25 ng of DNA. The thermal profile was 98°C for 30 s followed by 30 cycles of denaturation at 98°C for 10 s, annealing at 55°C or 61°C, depending on the primers, for 10 s, and extension at 72°C for 20 s, before ending with 72°C for 5 min and 4°C for 5 min. The purified PCR products were examined with gel electrophoresis in a 0.9% or 1.5% agarose gel containing 0.1 μg/ml ethidium bromide and visualized under UV light prior to sequencing (Institute of Biotechnology, University of Helsinki, Finland). Sequences were analyzed by comparing them to the NCBI GenBank database.

### 2.4 Bacterial strains and diet preparation

*Escherichia coli* OP50, which is used in *Caenorhabditis elegans* maintenance, was obtained from the Caenorhabditis Genetics Center (CGC). The bacteria were grown overnight in LB medium at 37°C before being seeded on NGM plates.

A wild-type curli protein producing *E. coli* strain MC4100 was used as positive control. Its mutant strain (LSR11) that is incapable of producing curli served as negative control. Both strains were kindly provided by Professor Matthew Chapman (University of Michigan, USA). Curli-producing *E. coli* was used as positive control, as it has been shown to induce alpha-syn aggregation in a *C. elegans* model of PD (Chen et al., 2016). Curli production was confirmed by growing the strains on Congo Red-YESCA (CR-YESCA) plates at 28°C for 48 hours. For the worm-feeding experiment, the strains were cultured in YESCA broth at 28°C for 48 hours. Next, 200 μl of the bacterial culture was seeded on NGM plates followed by incubation at 28°C for 48 hours. The plates were ready for use as nutrition for the worms.

Three *DSV* sp. isolates from PD patients (*D. desulfuricans*, *D. fairfieldensis*, *D. piger*) and three *DSV* sp. isolates from healthy individuals (*D. fairfieldensis* and two strains of *D. desulfuricans*) were used for worm-feeding experiments. The bacteria were cultured anaerobically for 4 days in Postgate broth at 37°C. Then, 5 ml of the bacterial suspension was centrifuged at 685 × g for 3 min to remove most precipitates. The bacterial cells were collected by an additional centrifugation at 7000 × g for 10 min, followed by resuspension in 1 ml of fresh Postgate broth. Bacterial concentration after these steps was approximately 10^6^ cfu/ml. Finally, 300 µl of the prepared *DSV* suspension was seeded on the center of NGM plates, which were then ready for the worms.

### 2.5 *Caenorhabditis elegans* growth conditions

*C. elegans* strain NL5901 producing human alpha-syn fused with yellow fluorescent protein (van Ham et al., 2008) was obtained from the CGC. The worms were grown on NGM plates seeded with *E. coli* OP50 at 20°C. Age synchronization was performed using the hypochlorite bleaching method (Stiernagle, 2006). The eggs were allowed to hatch overnight in M9 medium at room temperature with gentle agitation at 50 rpm. L1 larvae were then transferred to NGM plates seeded with *E. coli* OP50 and grown until L4 larvae stage at 20°C. At L4 stage, the worms were introduced to the eight prepared diets, including negative control *E. coli* LSR11, positive control *E. coli* MC4100, three *DSV* strains isolated from PD patients, and three *DSV* strains isolated from healthy individuals. The worms were then grown until day 4 adults. Worms were transferred to fresh plates every 2 days.

### 2.6 Confocal microscopy

Twenty worms per each diet condition were randomly selected and anesthetized in 10 µl of 50 mM levamisole hydrochloride (MP Biomedicals, Solon, OH, USA) placed on 3% agarose pads (five worms per pad). After one minute, the samples were covered with cover slips and sealed with CoverGrip™ Coverslip Sealant (Biotium, Fremont, CA, USA) to prevent the samples from drying. Z-stack images of the head region of the worms, which is from the tip of the mouth to right behind the pharyngeal terminal bulb, were taken at a high magnification (63x objective) with a Leica DM6 upright microscope (Leica Microsystems GmbH, Wetzlar, Germany). Mosaic images of each worm were merged and later used in image analysis.

### 2.7 Image analysis

Prior to image analysis with Imaris software version 9.7, the obtained images were converted to readable files with Imaris File Converter software. Fluorescent aggregates in the head region of the worms were quantified and measured using the automatic spot detection function. By using this function, the algorithm created spots covering the fluorescent aggregates. Thereby, the quantity and the volume of the fluorescent aggregates could be determined by quantifying and measuring these generated spots. The algorithm of the spot detection function was set by selecting “Segment only a region of interest”, which restricted the analysis to only the selected region (head of the worms), and “Different spot sizes (Region growing)”. Then, the XYZ diameter of the spots was typed in based on the estimated size of some fluorescent aggregates roughly measured in the Slices tab. Once the fluorescent aggregates were detected by the used algorithm, the quantity threshold was adjusted to best cover all fluorescent aggregates. For size determination, local contrast option was selected. The contrast area threshold was adjusted to best fit the sizes of the spots. Finally, background noises that were mistakenly selected by the algorithm were manually deleted. The statistics were exported to excel.

### 2.8 Survival assay

Adult worms were synchronized, and hatched L1 larvae were fed *E. coli* OP50 until L4 larvae stage at 20°C as described above. One hundred worms at L4 stage were then introduced to the eight prepared diets. They were transferred to fresh food and assessed for their survival every 2 days until day 4 adult stage. The number of survived and dead nematodes were recorded. Worms that did not respond to gentle touching and picking were considered dead and removed. Worms that were missing from the plates were excluded from the calculation. Experiments were performed in duplicates.

### 2.9 Statistical analysis

Statistical analysis was performed using IBM SPSS Statistics version 28.0. Shapiro-Wilk normality test was done for number and volumes of alpha-syn aggregates of individual bacterial strains (**Table S1**) and bacterial groups (**Table S2**). Kruskal-Wallis test was used to compare the quantity and the volume of alpha-syn aggregates between the worm groups (negative control, positive control, worms fed patient *DSV* strains and worms fed healthy *DSV* strains) and between worms fed distinct bacterial strains. Further pairwise comparisons were performed using Mann-Whitney U test. Kruskal-Wallis test was also used to compare the number of killed worms between feeding groups after 4 days being fed on the bacterial diets. A *P* value <0.05 was considered statistically significant. All tests were two-tailed.

## 3 Results

### 3.1 Detection of *Desulfovibrio* bacteria in fecal samples

For this study, fecal samples were collected from 10 patients with Parkinson’s disease and 10 healthy individuals. To know which fecal samples contain *DSV* bacteria, we performed conventional PCR with the species-specific primers and primers detecting [FeFe]-hydrogenase gene (*hydA*), which is present in nearly all *DSV* bacteria. As a result, all fecal samples of PD patients and eight fecal samples of healthy individuals were positive for *DSV* bacteria (**Table 2**). *D. fairfieldensis* was the most common *DSV* species detected in both groups.

**Table 2:** PCR detection of *Desulfovibrio* bacteria in the feces of PD patients and healthy individuals.

PD patient	Detected *DSV* species	*hydA*	Healthy individuals	Detected *DSV* species	*hydA*
**1**	*D. fairfieldensis*	+	I	*D. fairfieldensis*	+
**2**	*D. desulfuricans* *D. fairfieldensis*	+	II	None	None
**3**	*D. fairfieldensis*	+	III	*D. fairfieldensis*	+
**4**	*D. piger*	+	IV	*D. fairfieldensis*	+
**5**	*D. fairfieldensis*	+	V	*D. fairfieldensis*	+
**6**	None	+	VI	None	None
**7**	None	+	VII	None	+
**8**	*D. piger* *D. fairfieldensis*	+	VIII	*D. desulfuricans* *D. fairfieldensis*	+
**9**	*D. fairfieldensis*	+	IX	None	+
**10**	*D. fairfieldensis*	+	X	*D. fairfieldensis*	+

### 3.2 Isolation of *Desulfovibrio* bacteria

Fecal samples detected with *DSV* bacteria were used for bacterial isolation. *DSV* isolates were later used to feed *C. elegans* to assess alpha-syn aggregation. As a result, three *DSV* strains (*D. desulfuricans*, *D. fairfieldensis*, and *D. piger*) were isolated from the feces of three PD patients, and three *DSV* strains (*D. desulfuricans*, *D. desulfuricans* and *D. fairfieldensis*) were isolated from the feces of three healthy individuals. Bacteria from some *DSV*-positive samples failed to grow in the used conditions. In addition, pure cultures were not possible to obtain from some isolates, and thus, those strains could not be used in the study.

### 3.3 Quantification of alpha-syn aggregates in a *C. elegans* model

To assess the ability to induce alpha-syn aggregation of various *DSV* bacterial strains, a *C. elegans* model producing human alpha-syn fused with yellow fluorescent protein was used. The worms were fed with either *Desulfovibrio* strains isolated from feces of PD patients and healthy individuals, or *E. coli* strains as controls (positive control: curli-producing *E. coli* MC4100 and negative control: non-curli-producing *E. coli* LSR11). Alpha-syn aggregation was examined by confocal microscopy.

**Figure 1:**
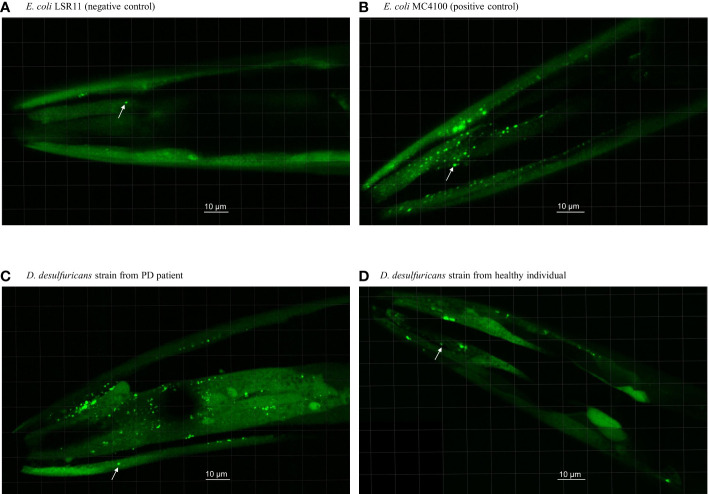
Head section of *C. elegans* worms fed with *E. coli* controls and *Desulfovibrio* bacteria from patients with Parkinson’s disease and healthy individuals. Worms were fed with **(A)** *E. coli* LSR11 (negative control), **(B)** *E. coli* MC4100 (positive control), **(C)** *D. desulfuricans* strain from PD patient and **(D)** *D. desulfuricans* strain from healthy individual. The arrows indicate some of the alpha-syn aggregates. The aggregates in worms fed with *DSV* bacteria from the PD patients were brighter, larger, and more abundant than the controls.

**Figure 2:**
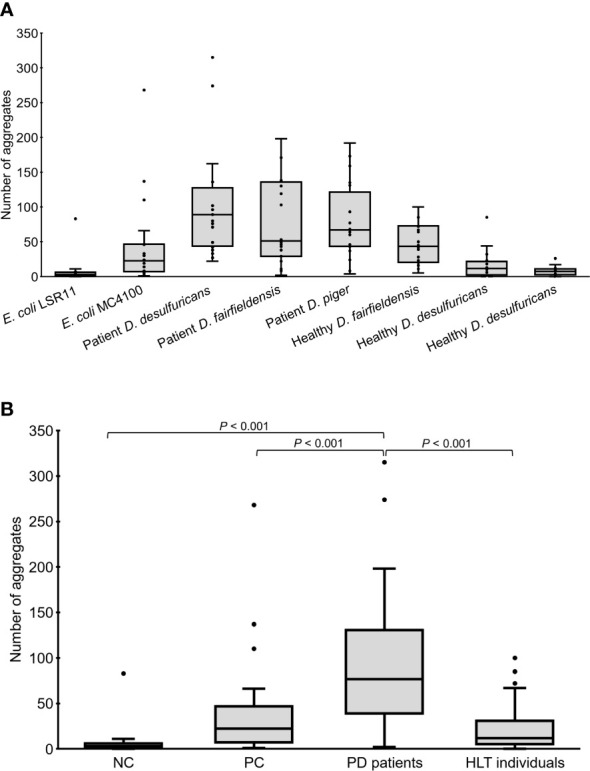
Number of alpha-syn aggregates in the head region of *C. elegans* at day 4 adult stage fed with different bacteria, demonstrated individually **(A)** or as groups **(B)**. NC: *E. coli* LSR11 (negative control), PC: *E. coli* MC4100 (positive control), PD patients: *DSV* strains from patients with Parkinson’s disease, HLT individuals: *DSV* strains from healthy individuals. Twenty worms per bacterial diet were analyzed. The dots represent the worms that were analyzed **(A)** or outliers **(B)**. The boxes represent the first and third quartile (lower and upper edges, respectively) and the medians (horizontal line within the boxes).

Worms fed *DSV* bacteria from PD patients had significantly more alpha-syn aggregates than worms fed *DSV* bacteria from healthy individuals or *E. coli* (76.5 vs. 12 or 3 or 22.5) (*P*<0.001, Kruskal-Wallis and Mann-Whitney U test) (**Figures 1**, **2**). On the other hand, worms fed *DSV* bacteria from healthy individuals had a quite similar number of aggregates as the positive controls (12 vs. 22.5) (*P*>0.05) and statistically more aggregates than the negative controls (12 vs. 3) (*P*<0.001). Within the group of worms fed *DSV* bacteria from PD patients, no significant difference in the quantity of alpha-syn aggregates was found between worms fed *D. desulfuricans, D. fairfieldensis*, or *D. piger* (**Figure 2A**).

### 3.4 Size determination of alpha-syn aggregates

**Figure 3:**
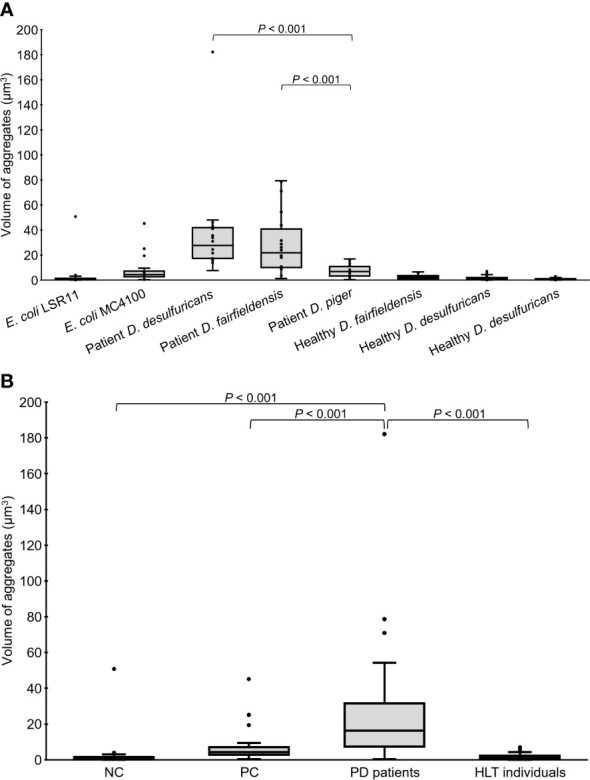
Volume of alpha-syn aggregates in the head region of *C. elegans* at day 4 adult stage fed with different bacteria, demonstrated individually **(A)** or as groups **(B)**. NC: *E. coli* LSR11 (negative control), PC: *E. coli* MC4100 (positive control), PD patients: *DSV* strains from patients with PD, HLT individuals: *DSV* strains from healthy individuals. Twenty worms per bacterial diet were analyzed. The dots represent the worms that were analyzed **(A)** or outliers **(B)**. The boxes represent the first and third quartile (lower and upper edges, respectively) and the medians (horizontal line within the boxes).

In addition to quantity, the volume of alpha-syn aggregates (µm^3^) accumulated in the head region of the worms was recorded. Statistical analysis revealed that the volume of alpha-syn aggregates in worms fed *DSV* bacteria from PD patients was significantly greater than that in worms fed *DSV* bacteria from healthy individuals (16.38 vs. 1.12), positive controls (16.38 vs. 4.27), or negative controls (16.38 vs. 0.76) (*P*<0.001, Kruskal-Wallis and Mann-Whitney U test) (**Figure 3**). Although worms fed *DSV* bacteria from healthy individuals harbored a considerable quantity of alpha-syn aggregates, the volume of the aggregates did not differ from that of negative controls (1.12 vs. 0.76) (*P*>0.05) and was clearly smaller than that of positive controls (1.12 vs. 4.27) (*P*<0.001). Within the group of worms fed *DSV* bacteria from PD patients, worms fed *D. desulfuricans* and *D. fairfieldensis* harbored statistically significantly larger alpha-syn aggregates than worms fed *D. piger* (27.67 and 21.79 vs. 6.76) (*P*<0.001) (**Figure 3A**). The alpha-syn aggregates of the largest size were found in worms fed *D. desulfuricans*.

### 3.5 Survival assay

**Figure 4:**
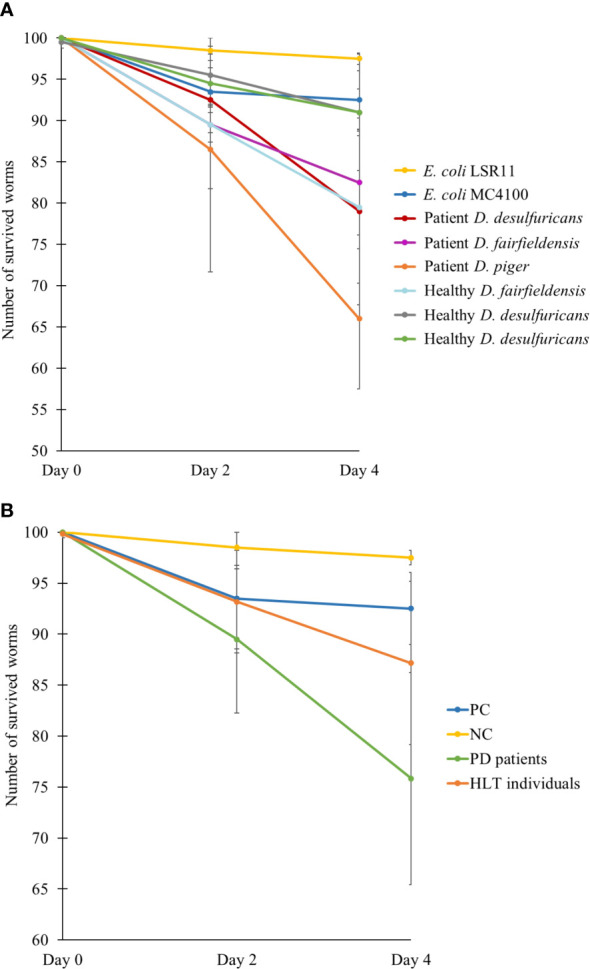
Number of survived worms after two and four days feeding on different bacteria, demonstrated individually **(A)** or as groups **(B)**. NC: *E. coli* LSR11 (negative control), PC: *E. coli* MC4100 (positive control), PD patients: *DSV* strains from patients with PD, HLT individuals: *DSV* strains from healthy individuals. Data are presented as mean ± SD.

To assess the effect of *DSV* bacteria on the survival of *C. elegans*, the number of live worms was counted after two and four days of feeding. After 2 days being introduced to the diets, the number of live worms was reduced more in the positive control and *DSV*-fed group than in the negative control (**Figure 4**). However, the positive control group had no further significant decrease in number while worms fed *DSV* bacteria continued to die. After 4 days, statistical analysis showed that worms fed *DSV* strains from PD patients died significantly more than worms in negative control group (24 ± 10 vs. 3 ± 1) (*P*<0.01, Kruskal-Wallis test).

## 4 Discussion

Aggregation of alpha-synuclein has been previously reported to be increased in the head region of nematodes and in the gut and brain of aged rats upon exposure to curli-producing *E. coli* (Chen et al., 2016). Correspondingly, peroral administration of curli-producing *E. coli* to mice overexpressing alpha-syn led to motor impairment and enhanced alpha-syn aggregation also in the gut and brain (Sampson et al., 2020). Here, we show that *DSV* strains, particularly those from PD patients, were more competent than curli-producing *E. coli* in stimulating the accumulation of larger and more abundant alpha-syn aggregates. Increased fatality was also observed in worms fed *DSV* bacteria, especially patient strains, possibly due to the unbearable amount of accumulated alpha-syn aggregates and different bacterial toxicity. Since *DSV* bacteria originating from healthy individuals also induced the aggregation of alpha-syn as compatible to curli-producing *E. coli*, we do not rule out the possibility that *DSV* bacteria, independently from the source, are capable of inducing alpha-syn aggregation. Taking into account that aggregation of alpha-syn is a hallmark of PD, the ability of *DSV* bacteria to induce alpha-syn aggregation in large numbers and sizes, as demonstrated in the present study, provides further evidence for the pathogenic role of *DSV* bacteria in PD, as previously suggested (Murros et al., 2021).

As an essential finding, all tested *DSV* bacteria induced alpha-syn aggregation in the head region of the *C. elegans* worm. Of further interest, *DSV* bacteria isolated from the fecal samples of PD patients were more competent to induce alpha-syn aggregation than the *DSV* bacteria isolated from the samples of healthy individuals. Furthermore, patient *DSV* strains significantly increased the fatality rate of the nematodes. These results suggest that besides common features that exist in all strains, *DSV* strains from PD patients appear to have greater virulence that enables them to have stronger toxicity and cause more alpha-syn aggregation. Although such virulence factors are unknown, some hypotheses, especially concerning the competence in H_2_S production, come into consideration. H_2_S has been reported to facilitate the release of cytochrome c from mitochondria to the cytoplasm in human cells (Baskar et al., 2007; Calenic et al., 2010). In addition, cytochrome c potentially triggers alpha-syn aggregation in the presence of reactive oxygen species (Hashimoto et al., 1999; Kumar et al., 2016). These findings favor the view that increased amounts of H_2_S producing bacteria such as *DSV* bacteria play a role in the pathogenesis of PD (Murros et al., 2021; Murros, 2022). Possibly, *DSV* strains differ more or less from each other concerning their effectiveness to produce H_2_S. H_2_S can be produced from sulfur-containing compounds in a catalase-mediated process (Olson et al., 2017). However, not all *DSV* strains owe catalase (Loubinoux et al., 2000) and presently the role of this enzyme in the functions of *DSV* bacteria has remained speculative. Notably, the hydrogenase system of *DSV* bacteria, which is composed of several enzymes of both [FeFe]- and [NiFe]-type, can vary considerably from one bacterial species to another (Baffert et al., 2019). As hydrogenases play a central role in donating electrons to the H_2_S-producing enzyme complex that consists of dissimilatory sulfate reductases (Singh and Lin, 2015), distinct differences in the hydrogenase systems may ultimately manifest as differences in the H_2_S production between different *DSV* strains.

Furthermore, the process leading to alpha-syn aggregation may depend on the varied ability of different *DSV* strains to interact with gastrointestinal cells. Various *DSV* strains have been shown to adhere to the epithelial cell surface, invade into the cell cytoplasm, and replicate within the cells (Bisson-Boutelliez et al., 2010). Interestingly, sulfate-reducing bacteria (SRB), to which group *DSV* bacteria belongs, have been shown to induce apoptosis of human colonic epithelial cells when the SRB enrichments originated from patients suffering from ulcerative colitis, whereas this response did not take place when SRB enrichments originated from healthy individuals (Coutinho et al., 2017). Taking these two studies into consideration, it is reasonable to presume that pathogenic *DSV* strains damage and impair intestinal barrier integrity and function. This would, firstly, lead to further *DSV* colonization of the human gut and allow interaction with surrounding cells, such as enteroendocrine cells, resulting in increased alpha-syn aggregation as proposed in the *DSV*-PD pathogenesis model (Murros et al., 2021). Secondly, damaged and dysfunctional intestinal barrier would eventually lead to intestinal inflammation. Inflammation in the intestine can be an underlying mechanism driving PD pathogenesis as it plays a part in the relationship between gut dysbiosis, immune responses and alpha-syn pathology (Houser and Tansey, 2017). Interestingly, gut inflammation reduces the capacity of H_2_S inactivation system (Flannigan et al., 2013), which allows *DSV*-produced H_2_S to accumulate and enhance alpha-syn aggregation. Last but not least, lipopolysaccharides which are known to be present in the outer membrane of Gram-negative bacteria, including *DSV* bacteria, may be an additional virulence factor. Lipopolysaccharides have been shown to modulate alpha-syn aggregation (Bhattacharyya et al., 2019) and enhance plasma H_2_S concentration in mice (Li et al., 2005). Of further interest, lipopolysaccharides of different *DSV* strains are diverse in structure (Zhang-Sun et al., 2015). The diversity can ultimately result in varied endotoxicity and different ability to induce alpha-syn aggregation.

Using a *C. elegans* model of PD, we observed that *DSV* bacteria enhanced alpha-syn aggregation in both size and quantity. In addition, we observed that *DSV* strains isolated from PD patients and healthy individuals had significantly different ability to induce alpha-syn aggregation and toxicity. The results indicate that *DSV* strains have different properties and those bearing particular pathogenic traits play a potential role in PD pathogenesis by inducing or accelerating alpha-syn aggregation. Future studies are necessary to further evaluate the role of these traits in disease development. As *DSV* bacteria are associated with PD and can induce alpha-syn aggregation, eradicating *DSV* bacteria or keeping their concentration at a low level could be a preventive strategy for PD. *DSV* strains isolated from PD patients and healthy individuals appear to have different traits, but it is not yet known how to differentiate between them except as presented in this study. Therefore, comparative genomics should be performed to identify genetic differences and pathogenic genes from PD patient *DSV* strains.

## Data availability statement

The original contributions presented in the study are included in the article, further inquiries can be directed to the corresponding author.

## Ethics statement

The studies involving human participants were reviewed and approved by Ethics Committee of Helsinki and the Uusimaa Health District (no. 2510/2020). The patients/participants provided their written informed consent to participate in this study.

## Author contributions

Conceptualization: VH, TT, KM, PS. Methodology: VH, TT, PS. Investigation: VH, BD. Formal analysis: VH. Resources: KM. Funding acquisition: PS. Project administration: PS. Supervision: VH and PS supervised BD, TT and PS supervised VH. Writing – original draft: VH. Writing – review & editing: VH, KM, TT and PS. All authors contributed to the article and approved the submitted version.
