# Supplementary material for: Desulfovibrio bacteria enhance alpha-synuclein aggregation in a Caenorhabditis elegans model of Parkinson’s disease
Source: Front Cell Infect Microbiol. 2023 May 1;13:1181315. doi: 10.3389/fcimb.2023.1181315 (PMC10183572; doi:10.3389/fcimb.2023.1181315)
Supplement: Supplementary file 1 [file DataSheet_1.pdf]

Supplementary Material

Desulfovibrio bacteria enhance alpha-synuclein aggregation in a *Caenorhabditis elegans* model of Parkinson's disease.

Vy A. Huynh, Timo M. Takala, Kari E. Murros, Bidhi Diwedi and Per E. J. Saris*

*** Correspondence:** Corresponding Author: per.saris@helsinki.fi

Supplementary Data

Table S1. Results of Shapiro-Wilk normality test for individual bacterial strains. LSR11: non-curli-producing *E. coli*, MC4100: curli-producing *E. coli*, MUU 26: patient *D. desulfuricans*, MUU 33: patient *D. fairfieldensis*, MUU 35: patient *D. piger*, MUU 34: healthy *D. fairfieldensis*, MUU 36: healthy *D. desulfuricans*, MUU 37: healthy *D. desulfuricans*.

Tests of Normality

		Kolmogorov-Smirnov[a]			Shapiro-Wilk		
	Bacterial_strains	Statistic	df	Sig.	Statistic	df	Sig.
N_of_aggregates	LSR11	.392	20	<.001	.370	20	<.001
	MC4100	.279	20	<.001	.658	20	<.001
	MUU 26	.244	20	.003	.804	20	<.001
	MUU 33	.220	20	.012	.908	20	.058
	MUU 34	.142	20	.200*	.932	20	.168
	MUU 35	.207	20	.024	.925	20	.122
	MUU 36	.243	20	.003	.723	20	<.001
	MUU 37	.125	20	.200*	.911	20	.067
V_of_aggregates	LSR11	.420	20	<.001	.322	20	<.001
	MC4100	.331	20	<.001	.645	20	<.001
	MUU 26	.311	20	<.001	.638	20	<.001
	MUU 33	.202	20	.033	.851	20	.006
	MUU 34	.187	20	.064	.876	20	.015
	MUU 35	.185	20	.070	.939	20	.225
	MUU 36	.260	20	.001	.765	20	<.001
	MUU 37	.274	20	<.001	.810	20	.001

*. This is a lower bound of the true significance.

a. Lilliefors Significance Correction

Table S2. Results of Shapiro-Wilk normality test for bacterial groups. HLT individuals: *DSV* strains from healthy individuals, NC: *E. coli* LSR11 (negative control), PD patients: *DSV* strains from patients with Parkinson's disease, PC: *E. coli* MC4100.

Tests of Normality

		Kolmogorov-Smirnov[a]			Shapiro-Wilk		
	Groups	Statistic	df	Sig.	Statistic	df	Sig.
N_of_aggregates	HLT individuals	.225	60	<.001	.779	60	<.001
	NC	.392	20	<.001	.370	20	<.001
	PD patients	.117	60	.041	.905	60	<.001
	PC	.279	20	<.001	.658	20	<.001
V_of_aggregates	HLT individuals	.178	60	<.001	.807	60	<.001
	NC	.420	20	<.001	.322	20	<.001
	PD patients	.202	60	<.001	.683	60	<.001
	PC	.331	20	<.001	.645	20	<.001

a. Lilliefors Significance Correction
